# Effects of bariatric surgery on testosterone level and sexual function in men with obesity: A retrospective study

**DOI:** 10.3389/fendo.2022.1036243

**Published:** 2023-01-24

**Authors:** Guoji Chen, Luping Sun, Shuwen Jiang, Xiaomei Chen, Jie Zhu, Xin Zhao, Shuqing Yu, Zhiyong Dong, Yuan Chen, Wen Zhang, Wah Yang, Cunchuan Wang

**Affiliations:** ^1^ Department of Metabolic and Bariatric Surgery, The First Affiliated Hospital of Jinan University, Guangzhou, China; ^2^ Jinan University Institute of Obesity and Metabolic Disorders, Guangzhou, China; ^3^ Joint Institute of Metabolic Medicine between State Key Laboratory of Pharmaceutical Biotechnology, The University of Hong Kong and Jinan University, Guangzhou, China; ^4^ Department of Gastrointestinal Surgery, The First People’s Hospital of Zhaoqing, Zhaoqing, China; ^5^ Department of urinary Surgery, First Affiliated Hospital of Jinan University, Guangzhou, China; ^6^ School of Nursing, Jinan University, Guangzhou, Guangdong, China

**Keywords:** sleeve gastrectomy (SG), gastric bypass, bariatric surgery, sexual function (male), obesity

## Abstract

**Introduction:**

Bariatric surgeries induce well-documented weight loss and resolve obesity comorbidities. Sexual function is one of the aspects of life quality and may benefit from surgery. Few studies have revealed the impact of bariatric surgeries on sexual function in Chinese men with obesity.

**Methods:**

This is a retrospective cohort study of patients undergoing bariatric surgery [laparoscopic sleeve gastrectomy (LSG) or laparoscopic Roux-en-Y gastric bypass (LRYGB)]. Data were collected between September 2017 and February 2022. The International Index of Erectile Function (IIEF) questionnaire was used to evaluate erectile function, intercourse satisfaction, orgasmic function, sexual desire, and overall satisfaction. Sex hormones and other blood tests were evaluated before and at least 1 year after the surgery.

**Results:**

Fifty-nine Chinese male patients completed the IIEF questionnaire. The multivariate logistic regression analysis revealed that body mass index (BMI) was the single independent risk factor of the severity of erectile dysfunction (ED). Preoperative testosterone levels had negative correlations with BMI and waist circumference. Thirty-seven patients completed the postoperative questionnaire with a mean follow-up of 23.2 months.

**Conclusion:**

BMI and waist circumference were negatively correlated with testosterone levels. BMI was an independent risk factor for the severity of ED. LSG and LRYGB led to positive and sustained improvement in sexual function of men with obesity. The two procedures had a comparable effect, more subjects being needed. Sex hormone levels also could be reversible. However, more weight loss did not predict a positive change in sexual function. A greater BMI loss might predict a greater increase in testosterone.

## 1 Introduction

The available data show a notably increasing trend in the prevalence of obesity in urban and rural China, where the estimated number rose from 37 million in 2004 to 85 million in 2018 (1). The population with obesity will likely keep increasing as Chinese economic society progresses. Both observational and high-quality randomized trials have shown that bariatric surgeries result in sustained weight loss and remission and improvement in type 2 diabetes mellitus (T2DM) and other obesity-related comorbidities, reducing the risks of microvascular and macrovascular complications (2–4). In an observational study, men with obesity were more likely to report erectile dysfunction (ED), reflecting hypogonadotropic hypogonadism (HH) (5). A multicenter cohort study has reported that nearly 74% of men with obesity were dissatisfied with their sex life (6). Sexual function is a private and sensitive topic not frequently discussed, correlating with patients’ well-being postoperatively. In addition to established outcome reporting standards for weight loss and comorbidities, there are validated instruments or questionnaires evaluating quality-of-life outcomes, including sex life, after bariatric surgeries (7). However, these standard instruments or questionnaires are not specifically focused on sexual function.

Recent studies demonstrated considerable improvement in sexual function after bariatric surgeries (8, 9). Considering cultural preferences, many people are reluctant to answer questions about sexual function in eastern countries (10). The impacts of bariatric surgery on sexual function in Chinese men with obesity were seldom studied. We conducted this retrospective study to explain the influence of obesity on sexual function and sex hormones, as well as the change in these perspectives after bariatric surgery.

## 2 Materials and methods

### 2.1 Ethics

Data of the study were extracted from the Chinese Obesity and Metabolic Surgery Database (COMES Database), which is endorsed by the Chinese Society for Metabolic and Bariatric Surgery (CSMBS). It is managed by the Chinese Obesity and Metabolic Surgery Collaborative (COMES Collaborative), which consists of bariatric surgeons, bariatric nurses, researchers, and healthcare professionals from 138 hospitals in China that increased gradually in the past 3 years. The study was approved by the Institutional Ethics Committee (KY-2022-131).

### 2.2 Study design

This is a retrospective cohort study. The cohort of patients at a single medical institution underwent laparoscopic sleeve gastrectomy (LSG) or laparoscopic Roux-en-Y gastric bypass (LRYGB) between September 2017 and February 2022. Follow-up time was defined as the time between the operation and the date of the last completion of the validated questionnaire for sexual function [International Index of Erectile Function (IIEF)]. All operations were performed by the same surgical team in our hospital. Standardized laparoscopic procedures were performed for all patients, which were described previously (11). Before the operation and on the follow-up day, patients were invited to finish the IIEF questionnaire on paper or online. Patients willing to participate in the study and completed the preoperative questionnaire were recruited for a follow-up visit at least 1 year postoperatively, including blood tests, anthropometric measurements, and re-collecting of their IIEF questionnaires. If a patient did not return to the hospital for our study, his most recent follow-up results were used for analysis and his blood test results from another hospital would be recorded in the COMES Database. The indication of surgeries and techniques were described previously (12, 13).

**Figure 1:**
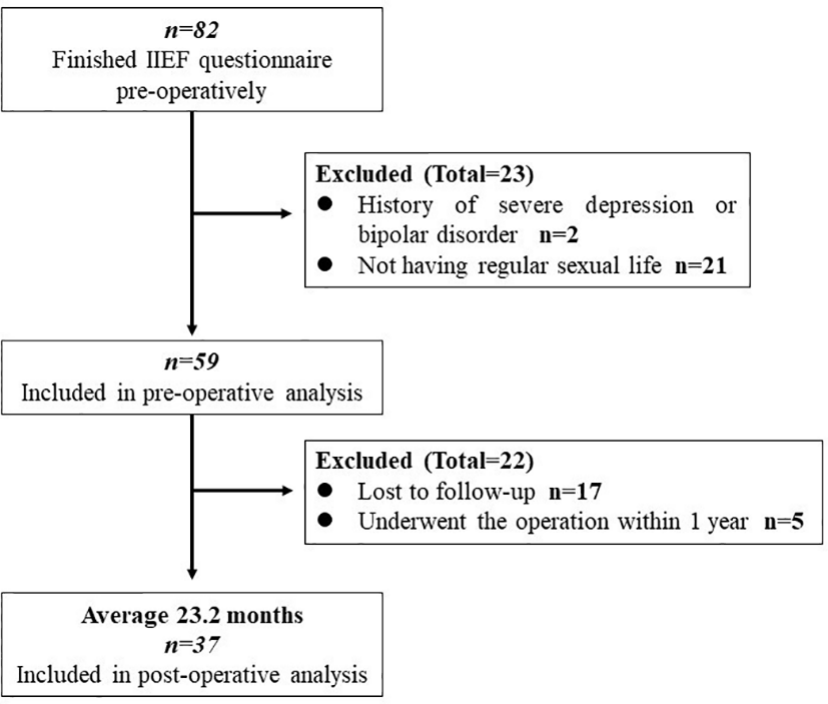
Study flow.

Inclusion criteria were as follows: men with obesity with ages ranging from 18 to 60 years; of Chinese nationality; patients who had regular sex lives. Patients finishing follow-up at least 1 year will be enrolled in the postoperative analysis. Exclusion criteria were as follows: patients with a history of psychiatric disease (severe depression and bipolar disorder), pituitary gland disease or hyperthyroidism, a habit of taking phosphodiesterase (PDE) inhibitors, e.g., sildenafil, and occurrence of mortality or major complications within 30 days after the operation (7). Once the patient chose “did not attempt intercourse” in the IIEF questionnaire, he would be excluded from the study. The study flowchart is shown in **Figure 1**.

Our primary outcomes were subjective feelings measured by the IIEF questionnaire and objective sex hormone test. Secondary outcomes were the weight loss effect and change in other blood test results.

Patients were asked to choose the answer to every item of the IIEF questionnaire at their direct feelings. Before bariatric surgeries, we did not give vitamin or microelement supplementation to the patients. After the operation, our team offered a multidisciplinary medically supervised weight loss program *via* Internet or telephone, which were composed of diet and behavioral and exercise advice, and pharmacotherapy advice would be available when necessary. Calcium tablet and multivitamin supplementations were recommended to the patients, which were described previously (14). All patients enrolled in the study were informed of the right to exit the study at any time. All of the data were stored and available in a prospective managed database (the COMES Database).

### 2.3 Anthropometric measurements and blood tests

Anthropometric measurements were collected before and after the operation as follows: neck circumference, breast circumference, waist circumference, hip circumference, weight, height, and body mass index (BMI). Blood tests were always collected at fasting state, including glycosylated hemoglobin (HbA_1c_), fasting plasma glucose (FPG), hemoglobin, C-reactive protein (CRP), vitamins, zinc (Zn), copper (Cu), iron (Fe), ferritin, hydroxyvitamin D [1, 25-(OH2) D3], total testosterone (TT), estradiol (E2), follicle-stimulating hormone (FSH), luteinizing hormone (LH), progesterone (PRO), prolactin (PRL), total cholesterol (TC), triglyceride (TG), high-density lipoprotein cholesterol (HDL-c), and low-density lipoprotein cholesterol (LDL-c), which were tested before surgery and at least 1 year after the operation. The percentage of excess weight loss (%EWL), percentage of total weight loss (%TWL), and percentage of excess BMI loss (%EBMIL) were calculated according to the standardized outcome reporting system (7).

### 2.4 Measures of sexual function

The IIEF is a widely used questionnaire with high sensitivity and specificity assessing men’s sexual function or erectile function (EF) concerned with medical treatment (15). Based on the syndrome of male sexual dysfunction, the IIEF questionnaire has been attested for its high quality and credibility in evaluating the efficacy of sexual ED therapies including sildenafil and other oral treatments (16–18). The usage of IIEF can be extended to diagnosing and assessing effectiveness in clinical monitoring (18, 19). As a result, total scores of IIEF and domain of EF scores are important. The 15-item questionnaire is composed of five domains: EF (items 1–5 and 15), intercourse satisfaction (IS; items 6–8), orgasmic function (OF; items 9 and 10), sexual desire (SD; items 11 and 12), and overall satisfaction (OS; items 13 and 14). The overall score of IIEF is 5–75, including 0–30 scores of EF, 0–15 scores of IS, 0–10 scores of OF, 2–10 scores of SD, and 2–10 scores of OS, respectively. Abnormal EF, also known as ED, is defined as scores of EF ≤25 (20). According to Sexual Health Inventory for Men (SHIM), scores of EF ranging from 22 to 25 are classified as mild ED, 17–21 as mild to moderate ED, 11–16 as moderate ED, ≤10 as severe ED (21).

### 2.5 Statistical analysis

Paired t-test was used to compare the preoperative and postoperative outcomes. We hypothesized that male patients’ IIEF scores improved by 10 beyond 1 year after the operation; the ratio of loss to follow-up was 30%, and the standard deviation was 10; 23 patients were needed to ensure a power of 95.0%, with a 2-sided alpha error of 0.05.

The normal distribution was attested *via* the Shapiro–Wilk normality test (P > 0.05). Two-sided P values <0.05 were considered statistically significant. Mean ± standard deviation was used for the description of continuous data, while median and percentile (25th and 75th) were used for non-normal distribution data. In order to identify potential risk factors correlated with scores of IIEF and EF domain, bivariate regression was applied. Univariate analysis was used for the recognition of potential predictors. Multivariate analysis including multiple linear regression or logistic regression was then used for further analysis. Paired t-test and two independent-samples t-tests were used if our data were distributed normally, and a nonparametric test was applied if the data were not normal. Chi-square test was used for categorical variables, and Fisher’s exact test would be applied for analysis when needed. The calculation of sample size was analyzed by PASS version 15.0 (NCSS Statistical Software, USA). Data were analyzed by Statistical Package for Social Sciences (SPSS), version 24.0 (SPSS Inc., USA). Heatmaps were constructed *via* GraphPad Prism version 8 (GraphPad Software, USA).

## 3 Results

### 3.1 Demographic data

Fifty-nine male patients with obesity were enrolled in our study, with a mean BMI of 40.37 ± 7.57 kg/m^2^ (ranging from 28.09 to 59.99 kg/m^2^) and mean age of 32.1 ± 6.7 years (ranging from 22 to 51 years). Seventeen of these patients were lost to follow-up visits. Five of them had undergone bariatric surgery in less than 1 year, not meeting our follow-up criteria. Consequently, 37 patients were involved in the postoperative analysis. No severe complication or mortality occurred in the first 30 days postoperatively.

In the studied patients, 50 (84.7%, 50/59) of them had scores that conformed to the ED definition. Thirty-one patients completed the IIEF questionnaire at least 1 year after the operation. Twenty-eight patients underwent LSG, while the other 31 patients underwent LRYGB. The mean follow-up was 23.2 months, ranging from 12 to 45 months. Demographic data were shown in **Table 1**.

**Table 1:** Demographic data of patients (n = 59).

	Mean ± SD	Min	Max
BMI (kg/m^2^)	40.31 ± 7.57	28.09	59.99
Age (years)	32.1 ± 6.7	22.0	51.0
Height (cm)	173.98 ± 6.53	159.30	188.00
Weight (kg)	122.12 ± 24.04	80.00	183.20
Neck circumference (cm)	45.9 ±4.4	32.0	56.0
Chest circumference (cm)	126.3 ± 11.3	99.5	154.0
Waist circumference (cm)	123.7 ± 11.2	83.5	170.0
Hip circumference (cm)	127.5 ± 15.4	93.5	162.0
Marital status (n, %)		Type of surgery (n, %)	
Married	36, 61.0%	LSG	28, 47.5%
Unmarried/divorced/widower	23, 39.0%	LRYGB	31, 52.5%
Educational level (n, %)		Severity of ED score (n, %)	
master’s degree or above	2, 3.6%	non-ED	9, 15.3%
bachelor’s degree	32, 54.2%	mild	24, 40.7%
college or below	25, 42.4%	mild to moderate	17, 28.8%
**Smoking (n, %)**	32, 54.2%	moderate	9, 15.3%
**Drinking (n, %)**	21, 35.6%	severe	0
Comorbidities (n, %)			
T2DM	25, 42.4%		
Hypertension	13, 22.0%		
Dyslipidemia	38, 64.4%		
OSA	25, 42.3%		

BMI, body mass index; T2DM, type 2 diabetes mellitus; OSA, obstructive sleep apnea; LSG, laparoscopic sleeve gastrectomy; LRYGB, laparoscopic Roux-en-Y gastric bypass; ED, erectile dysfunction.

### 3.2 Logistic regression analysis of preoperative International Index of Erectile Function (IIEF) and erectile function (EF) scores

Pearson’s correlation was used to identify potential predictors between preoperative IIEF scores and clinical and anthropometric variables. Only vitamin E was a positive factor related to preoperative IIEF scores (r = 0.283, P = 0.035).

**Figure 2:**
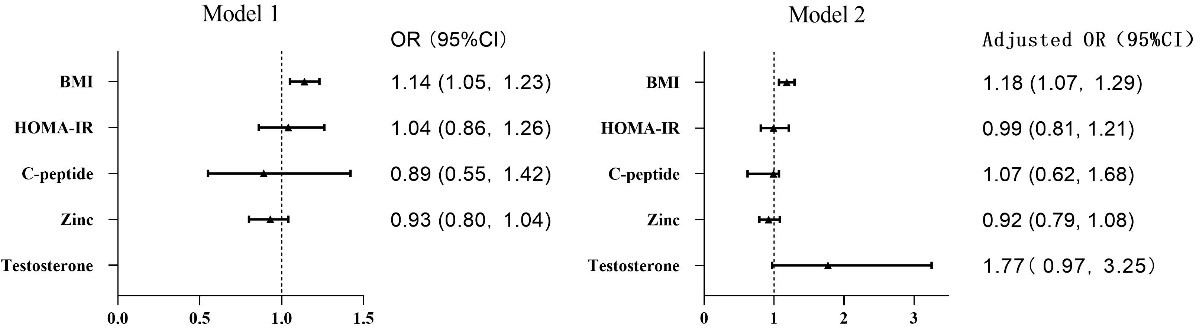
Forest plots of logistic regression analysis of preoperative IIEF and EF scores. BMI was the independent risk factor. BMI, body mass index; HOMA-IR, homeostasis model assessment of insulin resistance.

Univariate analysis showed that BMI, level of serum Zn, C-peptide, and HOMA-IR were predictive factors of preoperative ED grade. No significant difference was found in some important parameters, such as T2DM, hypertension, smoking, drinking, educational level, and marital status. The multivariate logistic regression analysis revealed that BMI was the single independent risk factor (OR = 1.14, 95% CI 1.05–1.23, P < 0.001). We adjusted for testosterone level, and BMI was still the independent risk factor. Details are presented in **Table 2** and **Figure 2**.

**Table 2:** Univariate and multivariate analyses of risk factors related to preoperative ED grade.

Univariate analysis			Multivariable logistic regression analysis			
			Model 1		Model 2	
Variable	F/ ^2^	P value	OR (95%CI)	P value	Adjusted OR	P value
T2DM	2.645	0.477*				
Hypertension	5.375	0.131*				
Marital status	1.908	0.604*				
Education level	9.649	0.078*				
BMI	4.277	0.017	1.14 (1.05, 1.23)	0.001	1.18 (1.08, 1.30)	<0.001
HbA1c	0.504	0.681				
Basal insulin	2.098	0.111				
Testosterone	1.320	0.295			1.77 (0.97, 3.25)	0.064
FSH	0.248	0.862				
LH	0.283	0.838				
Progesterone	0.550	0.651				
Prolactin	0.883	0.457				
Estradiol	1.383	0.259				
Zn	6.687	0.002	0.93 (0.80, 1.04)	0.373	0.92 (0.79, 1.08)	0.321
C-peptide	4.845	0.008	0.89 (0.55, 1.42)	0.612	0.99 (0.85, 1.16)	0.145
HOMA-IR	4.387	0.013	1.04 (0.86, 1.26)	0.660	0.99 (0.81, 1.21)	0.922

T2DM, type 2 diabetes mellitus; BMI, body mass index; HbA1c, glycosylated hemoglobin; Zn, zinc; HOMA-IR, homeostasis model assessment of insulin resistance Only significant or other important parameters are shown in the table. *Fisher’s exact test.

Model 2 was adjusted for testosterone level.

Test proportional odds assumption: ^2^ = 8.499, P = 0.386 (Model 1), ^2^ =9.840, P = 0.454 (Model 2).

Both the logistic regression models could get past the proportional odds assumption test (P > 0.05).

LH, luteinizing hormone; FSH, follicle-stimulating hormone.

### 3.3 Multiple linear regression for the preoperative level of testosterone

Pearson’s correlation showed that the preoperative testosterone level had significant relations with BMI, weight, neck circumference, breast circumference, waist circumference, hip circumference, vitamin A, vitamin E, albumin, HDL-c, and HOMA-IR. Blood test results of testosterone reflected the TT in the body. Considering that part of testosterone is combined with albumin and sex hormone-binding globulin (SHBG) in the blood and TT can be calculated from these parameters (22), non-negligible variance inflation factors (VIFs) existed between TT and albumin. Meanwhile, non-negligible VIFs also existed between BMI and weight and neck/chest/waist/hip circumference; hence, we constructed two multiple linear regression models. Preoperative testosterone levels had negative correlations with BMI (β = -0.047, P = 0.004) and waist circumference (β = -0.020, P = 0.005). Details are shown in **Table 3**.

**Table 3:** Correlation between preoperative testosterone level and anthropometric and clinical variables (n = 59).

Variable	Testosterone level		Multiple linear regression			
			Model 1		Model 2	
	r	P*	β	P	β	P
BMI	-0.451	< 0.001	-0.047	0.004		
Weight	-0.470	< 0.001				
Neck circumference	-0.457	0.001				
Chest circumference	-0.482	< 0.001				
Waist circumference	-0.548	< 0.001			-0.020	0.005
Hip circumference	-0.437	0.001			-0.093	0.684
Vitamin A	0.350	0.008	0.210	0.151	0.228	0.110
Vitamin E	0.282	0.033	0.144	0.322	0.142	0.336
Albumin	0.305	0.026				
HDL-c	0.315	0.015	0.143	0.277	0.137	0.299
Basal insulin	-0.232	0.076	0.083	0.613	-0.144	0.304
Constant			4.701		5.311	

P*, Pearson’s correlation; r: correlation coefficient.

HDL-c, high-density lipoprotein cholesterol.

### 3.4 Changes in IIEF scores and levels of sex hormones

#### Preoperation vs. postoperation

**Figure 3:**
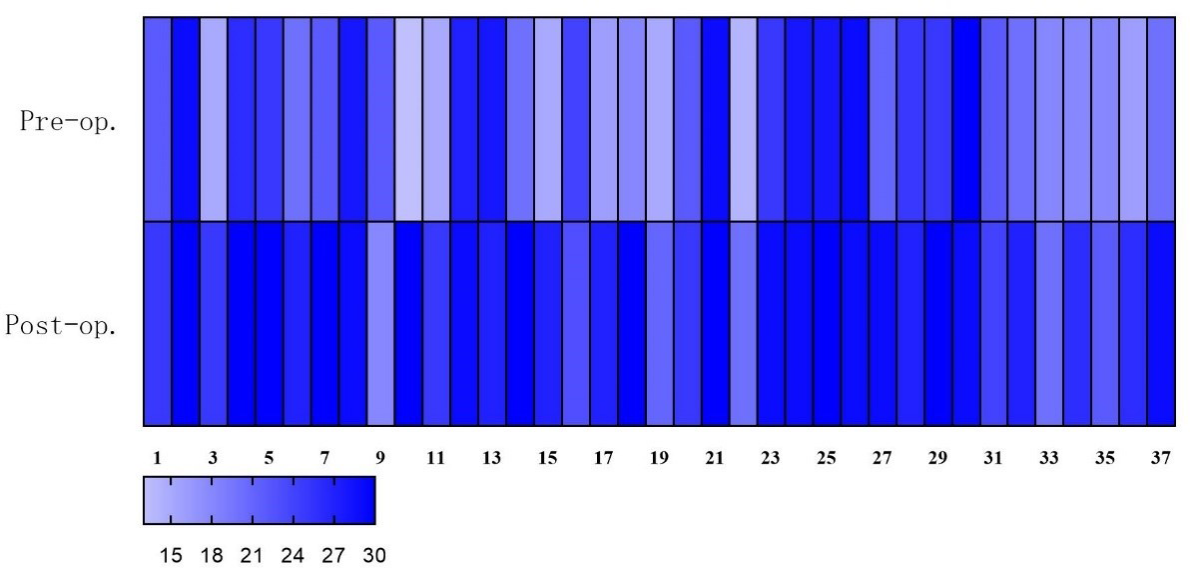
Heatmap: total scores of sexual function (IIEF scores) improved significantly postoperatively. Deeper color means higher scores.

**Figure 4:**
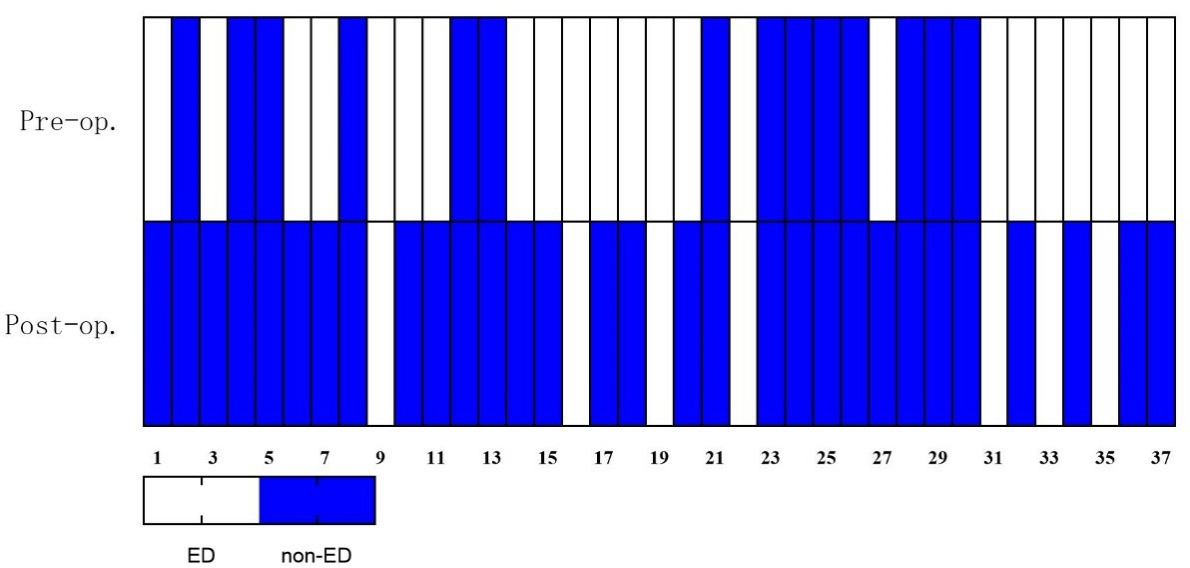
Heatmap: changes in EF domain based on erectile dysfunction classification. ED is diagnosed if EF scores ≤25. Patients with ED decreased from 27 (73.0%) to 11 (29.7%). EF, erectile function; ED, erectile dysfunction.

Eleven patients (29.7%, 11/37) had EF scores less than 25 at the last follow-up, but only two patients had slightly decreased scores of IIEF and EF domain, while the others showed increased scores. Overall, there were significant improvements in scores of IIEF (preoperation 52.22 ± 10.79 vs. postoperation 61.46 ± 7.93, P < 0.001) and its domains in the patients, meaning that they were more satisfied with their EF and sex life postoperatively. Changes in IIEF scores were shown *via* heatmaps (**Figures 3**, **4**). In the postoperative sex hormonal profile measurement, patients’ testosterone levels also significantly rose from 2.87 ± 1.00 ng/ml to 5.45 ± 0.84 ng/ml (P < 0.001). Serum FSH and LH increased significantly. The decrease in prolactin and estradiol reached statistical significance. Data from the questionnaire were presented in **Table 4**. With excellent weight loss, HbA1c, FPG, basal insulin, HOMA-IR, and triglyceride significantly decreased. Ferritin significantly decreased, but none reached the standard of deficiency. Several patients who lacked vitamin B2 and B12 were advised regular supplements. In **Table 5**, alterations of sex hormones and other important clinical and anthropometric parameters are shown in detail.

**Table 4:** Changes of IIEF score and its five domains.

	Preoperation (n=59)		Postoperation (n=37)		P value*
	Mean ± SD	Median (IQR)	Mean ± SD	Median (IQR)	
IIEF	52.22 ± 10.79	50 (43.5, 62)	61.46 ± 7.93	61 (56, 67.5)	<0.001
Erectile function	21.84 ± 5.08	22 (18, 26.5)	26.86 ± 3.35	27 (25, 30)	<0.001
Orgasmic function	7.76 ± 1.85	8 (6.5, 9.5)	9.00 ± 1.27	9 (8.5, 10)	<0.001
Sexual desire	6.51 ± 1.90	7 (5, 8)	7.43 ± 1.41	7 (6.5, 8)	0.008
Intercourse satisfaction	9.03 ± 2.80	9 (6, 11)	10.51 ± 2.52	11 (8, 12.5)	0.001
Overall satisfaction	6.92 ± 1.98	7 (6, 8)	7.68 ± 1.81	8 (6.5, 8.5)	0.034

IIEF, International Index of Erectile Function.

P value*: paired t-test.

**Table 5:** Changes in anthropometric and clinical variables before and after bariatric surgery.

Variable	Preoperation (n=59)			Postoperation (n=37)			P value*
	Mean	SD	Range	Mean	SD	Range	
Anthropometric measurements							
BMI (kg/m^2^)	41.31	7.57	28.09-59.99	27.78	4.24	19.79-36.11	<0.001
Weight (kg)	122.12	24.04	80.00-183.20	84.33	15.48	58.00-120.00	<0.001
%EWL				85.0	37.6	8.6-199.7	
%TWL				30.3	14.1	1.6-59.6	
%EBMIL				88.0	36.3	23.7-198.7	
Waist circumference (cm)	123.7	18.2	83.5-170.0	101.9	15.3	75.5-130.0	<0.001
Hip circumference (cm)	127.5	15.4	93.5-162.0	106.6	11.9	92.5-135.5	<0.001
Sex hormones							
Testosterone (ng/ml)	2.87	1.00	1.22-5.93	5.45	0.84	3.97-7.2	<0.001
Estradiol (pg/ml)	42.62	27.22	10.50-155.48	32.58	11.90	12.00-55.35	0.036
Prolactin (ng/ml)	11.59	5.95	4.51-40.95	8.33	4.45	0.20-21.41	<0.001
Progesterone (ng/ml)	0.49	0.51	0.05-3.58	4.45	8.34	0.20-4.28	0.310
LH (mIU/ml)	4.54	2.08	1.64-12.11	5.66	1.36	2.89-8.67	0.005
FSH (mIU/ml)	5.21	2.82	1.29-13.58	6.73	2.97	2.25-16.73	<0.001
Blood glucose metabolism, hemoglobin, and CRP							
HbA1c (%)	6.78	1.76	4.80-11.60	4.23	0.17	4.3-6.3	<0.001
Basal insulin (mIU/L)	22.33	13.09	4.58-73.71	7.03	5.56	1.37-29.32	<0.001
FPG (mmol/L)	7.17	2.77	4.10-14.84	5.13	0.79	4.04-27.20	<0.001
HOMA-IR	5.52	10.29	5.25-50.59	0.82	1.05	0.10-4.70	0.010
Hemoglobin (g/L)	154.99	11.75	127.70-179.00	150.40	12.00	122.00-173.00	0.082
CRP (mg/L)	8.85	11.49	0.70-71.82	2.52	4.38	0.24-21.23	0.008
Vitamins and microelements							
Vitamin A (nmol/L)	1.33	0.53	0.40-2.25	1.36	0.47	0.42-2.31	0.204
Vitamin B1 (nmol/L)	70.43	19.02	45.25-129.09	70.08	17.01	45.98-111.62	0.478
Vitamin B2 (ng/L)	331.50	58.58	196.96-498.32	292.73	56.96	197.00-422.67	0.004
Vitamin B6 (nmol/L)	31.02	15.70	11.25-61.91	30.25	11.14	12.42-53.92	0.793
Vitamin C (nmol/L)	51.58	13.95	31.07-85.58	56.65	17.15	30.51-96.62	0.187
Vitamin E (ng/ml)	13.12	1.88	9.54-16.84	12.85	1.85	9.33-16.16	0.727
1, 25-(OH_2_) D_3_ (ng/ml)	16.14	6.46	4.69-35.90	35.20	65.41	6.74-360.05	0.116
Vitamin B12 (pg/ml)	414.08	213.65	211.48-1244.86	282.78	187.87	2.72-736.08	0.002
Ferritin (ng/ml)	283.72	178.40	13.75-1082.88	171.23	101.67	26.42-389.04	<0.001
Fe (μmol/L)	17.71	6.23	7.70-36.20	20.68	6.58	11.50-37.10	0.051
Zn (μmol/L)	9.57	3.33	4.90-23.00	9.23	3.87	4.47-27.20	0.790
Cu (μmol/L)	15.96	3.70	10.10-26.60	15.10	4.42	2.10-25.50	0.172
Lipid metabolism							
Total cholesterol (mmol/L)	5.07	1.24	1.12-8.95	4.65	0.80	2.03-7.37	0.168
Triglyceride (mmol/L)	2.58	1.88	0.58-8.84	1.45	0.96	0.50-4.61	0.001
HDL-c (mmol/L)	1.09	0.50	0.64-3.09	1.16	0.62	0.59-1.73	0.788
LDL-c (mmol/L)	3.02	0.79	0.96-5.22	2.63	0.59	1.49-4.46	0.053

HOMA-IR, homeostasis model assessment of insulin resistance; 1, 25-(OH2) D3, 1, 25 - dihydroxy - vitamin D; Fe, iron; Zn, zinc; Cu, Copper *:paired t-test.

%EWL, percentage of excess weight loss; %TWL, percentage of total weight loss; %EBMIL, percentage of excess BMI loss; FPG, fasting plasma glucose; LH, luteinizing hormone; FSH, follicle-stimulating hormone; CRP, C-reactive protein; HDL-c, high-density lipoprotein cholesterol; LDL-c, low-density lipoprotein cholesterol.

### 3.5 LSG vs. LRYGB

We tried to compare the effect of LSG and LRYGB on sexual function. There was no significant difference in baseline and weight loss between the LSG and LRYGB groups. A significant difference in IIEF and its domains was not found in the two independent-sample t-tests. A difference in the levels of testosterone was not found either. LSG and LRYGB exerted similar and positive effects on sexual function. Details are shown in **Table 6**.

**Table 6:** Outcomes of bariatric surgery and IIEF scores of the two groups after bariatric surgery.

	LSG	LRYGB	P value
Subjects (n)	18	19	
Preoperative BMI	41.98 ± 7.89	40.32 ± 8.02	0.530
Preoperative age	30.8 ± 7.3	32.8 ± 6.9	0.407
Preoperative T2DM	8	8	0.887
Preoperative hypertension	2	5	0.231*
%TWL	28.57% ± 10.79%	31.84% ± 16.83%	0.484
%EWL	85.79% ± 30.76%	84.21% ± 43.94%	0.901
%EBMIL	89.52% ± 27.87%	86.36% ± 44.59%	0.813
Postoperative IIEF	62.67 ± 8.89	60.32 ± 6.95	0.375
Erectile function	27.28 ± 3.20	26.47 ± 3.53	0.473
Orgasmic function	8.94 ± 1.35	9.05 ± 1.22	0.800
Sexual desire	7.61 ± 1.54	7.26 ± 1.28	0.459
Intercourse satisfaction	11.22 ± 2.71	9.84 ± 2.19	0.097
Overall satisfaction	7.67 ± 2.33	7.68 ± 1.20	0.977
Postoperative testosterone	4.99 ± 0.79	5.26 ± 0.90	0.552

*:Fisher’s exact test; the others were two independent-samples t-test. Scores of IIEF questionnaire: mean ± standard deviation.

LSG, laparoscopic sleeve gastrectomy; LRYGB, laparoscopic Roux-en-Y gastric bypass; %TWL, percentage of total weight loss; %EWL, percentage of excess weight loss; %EBMIL, percentage of excess BMI loss; IIEF, International Index of Erectile Function.

Finally, we aimed to determine predictive factors of sexual function improvement and testosterone improvement (ΔIIEF, ΔEF, and Δtestosterone). Potential relationships were explored between postoperative scores of IIEF or postoperative level of testosterone and all parameters above, including ΔBMI, %EWL, %TWL, and %EBMIL. Still, none of the calculations reached statistical significance. However, there was a tendency that with greater BMI loss, testosterone level increased more (r = -0.307, P = 0.065). Univariate analysis is shown in **Table 7**.

**Table 7:** Univariate analysis of the change in IIEF scores, EF scores, and testosterone level.

	ΔIIEF		ΔEF		ΔTestosterone	
	r	P	r	P	r	P
ΔBMI	-0.123	0.470	-0.123	0.470	-0.304	0.065
EWL	-0.037	0.826	-0.140	0.410	0.073	0.668
TWL	0.182	0.282	0.100	0.555	0.055	0.748
EBMIL	0.189	0.262	0.135	0.425	0.055	0.745

BMI, body mass index; %TWL, percentage of total weight loss; %EWL, percentage of excess weight loss; %EBMIL, percentage of excess BMI loss; IIEF, International Index of Erectile Function; EF: erectile function. The change in IIEF scores, EF scores, and testosterone level were defined as: (postoperative data minus preoperative data). We did not find predictors among weight loss effect and other indexes, but it showed a tendency that with more BMI loss, testosterone level increased more.

## 4 Discussion

The main results of our study demonstrated the worsening and dominating impacts of obesity on sexual dysfunction, emphasizing the impressive postsurgical effects on sexual function in the studied group of the Chinese population. Sexual function was evaluated by IIEF scores, and a comparison of the two main surgical procedures (LSG and LRYGB) was presented. In the background of the conservative and traditional culture in China, it is rare and even a taboo to discuss sex life. Many patients did not have regular sex lives and were not willing to participate in our study. These reasons limited our sample size. Since most other related studies recruited a similar sample size and reported the outcome of a half year or 1 year postoperatively, our study showed that bariatric surgeries had sustained and favorable impacts on sexual function, testosterone level, and comorbidities with a longer follow-up.

### 4.1 Correlations of sexual dysfunction

Arolfo et al. (23) found a negative Spearman’s correlation between preoperative serum Zn and quality-of-life scores. Microelements in men with obesity may have relationships with sexual function. Interestingly, our univariate analysis of ED grade also displayed statistical significance between Zn and ED grade. Apart from Zn, we found that vitamin E had a positive correlation with preoperative IIEF scores. Vitamin A and vitamin E had positive relations with the preoperative level of testosterone. Obesity results in oxidative stress, while vitamins A and E are antioxidants in the body, and antioxidant treatment, including vitamins in men, can increase serum sex hormone levels (24). Zn participated in the synthesis of testosterone. But which microelement can best reflect sexual function is unclear. Multifactorial mechanisms may be involved.

Sarhan et al. (8) found that weight was the factor predicting scores of preoperative IIEF, and both age and BMI predicted preoperative testosterone levels. It had been estimated that one-third of men with obesity, T2DM, or metabolic syndrome have subnormal free testosterone concentrations and HH (25, 26). But in our study, only vitamin A level correlated significantly with preoperative IIEF scores. Higher BMI and higher waist circumference were correlated with a lower testosterone level. Mechanisms may include that excess body fat plays a fundamental role in inflammation status, leading to a decreased level of gonadotropin and testosterone (25, 27).

Few other studies tried to find out the relationships between ED grade and parameters. Analyses of our data were focused on ED grade, showing that BMI was the independent risk factor and that a higher BMI led to more apparent syndromes of ED. Secondary T2DM and hypertension may not contribute very adversely on erectile or sexual function. But with younger age than most samples of articles focused on bariatrics and sexual function, short durations of comorbidities (many of these comorbidities were diagnosed for the first time before the operation) might not take the lead.

### 4.2 Impact of bariatrics on sexual function

With the mean %EWL over 80% after bariatric surgeries, IIEF scores and its five domains significantly increased. Total scores of IIEF rose to 61.46 ± 7.93, which were similar to those of the normal control group of Rosen et al. (15) in their establishing procedure of IIEF and to other articles concentrated on sexual function influenced by bariatrics (28). Our result is similar to those of prior articles that all domains increased (8, 23), while some articles did not show a significant increase in every domain (29, 30). Although there were still 11 patients with EF domain scores ≤25, nine of them improved compared with preoperative scores. A study reported significant improvements in EF measured by the simplified version of IIEF (IIEF-5) in 39 men with obesity and ED 1 year following RYGB (31). IIEF-5 is a questionnaire assessing EF, and IIEF can evaluate sexual function from multidimensional scales. At the same time, there was an increasing marginal significance in testosterone level (P = 0.052) (31). Taskin et al. (32) revealed that patients with T2DM (mean age = 51.5 ± 9.3 years, mean BMI = 34.9 ± 3.8 kg/m^2^) experienced a mean increase of testosterone level of 1.4 ± 1.2 ng/ml 6 months after sleeve gastrectomy with transit bipartition (SG-TB). Our results showed significant and sustained improvements in sex hormones, reversing the HH trend. Testosterone level rose from 2.87 ± 1.00 ng/ml to 5.45 ± 0.84 ng/ml. The advances in sexual function were accompanied by favorable changes in metabolic conditions. Recent studies applying the IIEF questionnaire revealed that bariatric surgeries positively impacted sexual function in men with obesity 1 year postoperatively in different countries (8, 21, 23, 29, 30).

Mora et al. (33) found that ΔBMI had positive correlations with ΔIIEF and ΔEF, concluding that weight loss is the major contributor to improved sexual function. Taskin et al. (31) found that ΔBMI predicted the change in testosterone level. We have tried to establish a statistical model to find out predictors of improving the degree of sexual function (ΔIIEF scores), EF (ΔEF scores), and testosterone level (Δtestosterone). ΔTestosterone level seemed to have a correlation with BMI loss. This result might be limited by the sample size. However, no statistical significance was found between these parameters and the weight loss effect (ΔBMI, %EWL, %TWL, and %EBMIL). This may be due to effects of other factors after surgery. Playing a dominant role in the impairment of sexual function and testosterone level, a higher BMI led to decreased testosterone, chronic inflammation and oxidative stress, insulin resistance and T2DM, hypertension, drug usage, excess fat covering genitalia, and psychological factors. In combination, these factors impair sexual function (27, 34, 35). With good weight loss after surgery, these negative influences gradually disappeared and metabolic abnormality improved. Then, the sexual function and sex hormone levels ameliorated.

A previous article reported a negative impact on sexual function. With a mean follow-up of 31.8 months, laparoscopic adjustable gastric banding did not maintain sufficient weight loss (mean BMI loss was 7.51 kg/m^2^, while in our study, it was 13.35 kg/m^2^), and a worsening trend of scores in IIEF and EF appeared (36). This outcome demonstrated that unfulfilled weight loss might cause worse EF. The two objects of our study having decreased EF scores had %EWL >50%, showing better testosterone levels and other indexes. As **Table 7** illustrated, patients did not suffer from evident deficiency of vitamins or microelements.

Apart from the factors discussed above, sexual function is affected by relationships with the lover. As Gokalp et al. (28) pointed out, along with improvements in male patients’ IIEF scores, their partners became more pleased with their sex lives. Couples were able to benefit from LSG from another perspective. In addition, IIEF cannot assess fertility and premature ejaculation. Fertility outcome was not included in our study. However, Fariello et al. (37) revealed that testicular oxidative stress decreased after weight loss 6 months postoperatively, and both seminal quality and sex hormone profile improved.

### 4.3 Effects of LSG and LRYGB

LSG and LRYGB, the most widely used bariatric procedures worldwide and in China (38, 39), nearly reached the ratio of 1:1 in our study. A single operative procedure was incorporated within almost all articles previously published. El-Tholoth et al. (21) compared the proportion of men with ED after LSG and LRYGB, with more mild-to-moderate ED being found in the LRYGB group (LSG n = 0 vs. LRYGB n = 2). Mild ED and non-ED were not different in proportion. Our center found that the two procedures exerted a positive and comparable influence on sexual function evaluated by the IIEF questionnaire. The two groups did not significantly differ in EF and satisfaction with their sex lives. Still, the deficiencies of vitamins, microelements, and hemoglobin in the 19 patients undergoing LRYGB were not found during their follow-ups. It should also be noted that more samples are needed to prove this non-inferior effect. The lack of subjects made the result only hypothetical.

### 4.4 Strengths and limitations

The limitation of this retrospective study lies in the data size, keeping us from considering more socioeconomic factors. Two reasons limited the sample size. Firstly, many patients with obesity were not in a romantic relationship or not married, not having a regular sex life, which explains why our study and similar studies include only tens of subjects. Secondly, the number of men with obesity undergoing bariatric surgeries is several times smaller than that of women as a matter of experience in our center. We could not obtain a consecutive data series because it is inconvenient for patients to regularly travel long journeys back to our hospital from different cities. This can be resolved by future multicenter studies in which patients can be regularly evaluated nearby. With a mean follow-up period longer than that of many other studies, significant improvements in sex life and sex hormones were observed in this study. To the best of our knowledge, this is the first report on the sexual function of men with obesity before and after bariatric surgery in China. Also, limited data were found in Asia. Consequently, our data can bring about some new evidence. Our retrospective study might be the start of a multicenter study from which we can get a more comprehensive view of the sex lives of men with obesity undergoing bariatric surgery. In addition to the results above, fertility and correlations between sexual ability and other indices are expected.

## 5 Conclusions

BMI and waist circumference were negatively correlated with the level of testosterone. BMI was an independent risk factor for the severity of ED. LSG and LRYGB led to positive and sustained improvement in sexual function of men with obesity. The two surgical procedures had a comparable effect; more subjects are needed. Impaired sex hormone levels also could be reversible. The mechanism might be concerned with many factors, as the degree of weight loss effect and other indexes could not predict the improving degree of scores of sexual function. A greater BMI loss might predict a greater increase in testosterone.

## Data availability statement

The original contributions presented in the study are included in the article/supplementary material. Further inquiries can be directed to the corresponding authors.

## Ethics statement

The studies involving human participants were reviewed and approved by IRB of the First Affiliated Hospital of Jinan University (KY-2022-131). The patients/participants provided their written informed consent to participate in this study. Written informed consent was obtained from the individual(s) for the publication of any potentially identifiable images or data included in this article.

## Author contributions

All authors listed have made a substantial, direct, and intellectual contribution to the work and approved it for publication.
